# Redox-Responsive π-Conjugated Prodrug Nanoassemblies for Cancer Chemotherapy

**DOI:** 10.3390/pharmaceutics17091162

**Published:** 2025-09-04

**Authors:** Shuwei Liu, Liuhui Chen, Hongyuan Zhang, Yuequan Wang, Cong Luo

**Affiliations:** 1 Department of Pharmaceutics, Wuya College of Innovation, Shenyang Pharmaceutical University, Shenyang 110016, China; liushuwei201@gmail.com (S.L.); chenyao5818@gmail.com (L.C.); zhanghongyuan202@gmail.com (H.Z.); 2 Joint International Research Laboratory of Intelligent Drug Delivery Systems of Ministry of Education, Shenyang Pharmaceutical University, Shenyang 110016, China

**Keywords:** molecular design, doxorubicin prodrug, nanoassembly, drug delivery, drug release

## Abstract

**Background**: Redox-responsive prodrug nanoassemblies (NAs) have been extensively utilized in precise cancer therapy. But there is no research shedding light on the impacts of the π–π stacking interactions on the self-assembly capacity of redox-responsive prodrugs and the in vivo delivery fate of NAs. **Methods**: Three structurally engineered doxorubicin (DOX) prodrugs (FAD, FBD, and FGD) were developed through α-, β-, and γ-positioned disulfide linkages with π-conjugated Fmoc moieties. The NAs were comprehensively characterized for their self-assembly kinetics, redox-responsive drug release profiles, and physicochemical stability. Biological evaluations included cellular uptake efficiency, in vivo pharmacokinetics, and antitumor efficacy in tumor-bearing mouse models. **Results**: Systematic characterization revealed that π-conjugated disulfide bond positioning dictates prodrug self-assembly and inversely regulates reductive drug release relative to carbon spacer length. The FBD NAs demonstrated optimal redox-responsive release kinetics while maintaining minimal systemic toxicity, achieving 101.7-fold greater tumor accumulation (AUC) than DiR Sol controls. In 4T1 tumor-bearing models, FBD NAs displayed potent antitumor efficacy, yielding a final mean tumor volume of 518.06 ± 54.76 mm^3^ that was statistically significantly smaller than all comparator groups (*p* < 0.001 by ANOVA at a 99% confidence interval). **Conclusion**: These findings demonstrate that strategic incorporation of redox-sensitive disulfide bonds with different π–π stacking interactions in the prodrug structure effectively optimizes the delivery-release balance of DOX in vivo, ensuring both potent antitumor efficacy and reduced systemic toxicity.

## 1. Introduction

Cancer remains a leading global health burden, with limited therapeutic efficacy and debilitating complications [1,2,3]. Conventional therapeutic approaches include surgery and chemotherapy, with evidence demonstrating that perioperative chemotherapy combined with surgery markedly improves local tumor control and overall survival compared to surgery alone [4]. Notably, chemotherapy remains indispensable for treating metastatic tumors unsuitable for surgical resection [5,6]. Doxorubicin (DOX) is a first-line, broad-spectrum chemotherapeutic agent that exerts its antitumor effects primarily through interference with DNA and RNA synthesis and inhibition of topoisomerase II. [7,8,9]. While demonstrating remarkable efficacy against various solid tumors (e.g., breast, lung, and ovarian cancers) and hematological malignancies (e.g., acute leukemia and lymphoma), DOX administration is hampered by severe toxicities [10,11]. The aqueous formulation exhibits dose-limiting cardiotoxicity, manifesting as arrhythmias and heart failure, creating a narrow therapeutic window that restricts clinical utility [10]. The 1995 FDA approval of PEGylated liposomal DOX (Doxil^®^) marked a milestone in nanomedicine. This formulation utilizes an ammonium sulfate gradient loading method to encapsulate DOX crystals within liposomes [12], demonstrating dual therapeutic advantages: enhanced tumor accumulation through the Enhanced permeability and retention (EPR) effect coupled with reduced cardiac distribution, along with prolonged circulation half-life achieved via PEG-mediated evasion of reticuloendothelial system clearance [13]. Despite these improvements, Doxil^®^ had long been criticized for unsatisfactory clinical translation, inefficient drug release due to rigid lipid bilayers, and potential biosafety risks of carrier materials [14,15]. These unresolved issues underscore the urgent need for novel drug delivery systems that optimize the balance between efficacy and safety in clinical chemotherapy.

To overcome the limitations of conventional carrier-based nanomedicines, carrier-free nanoassemblies composed of small-molecule drugs or prodrugs have emerged as a promising platform for cancer therapy [16]. These systems retain the advantages of traditional nanomedicines while offering distinct benefits, including simple fabrication, high drug loading (>50%), and the elimination of carrier-related toxicity [17,18]. In particular, prodrug-based nanoassemblies integrate the precision of prodrug design with the efficiency of nanoscale delivery, representing a paradigm shift in drug development [19]. An ideal prodrug strategy involves transient inactivation of the parent drug via chemical modification, followed by selective reactivation at disease sites, thereby minimizing off-target effects and enhancing therapeutic specificity [20,21]. Tumor-specific activation is enabled by the abnormal chemical milieu of tumors, such as elevated levels of reactive oxygen species (ROS) and glutathione (GSH) [22,23]. Accordingly, researchers have engineered cleavable linkers—such as disulfide bonds—that respond to these cues and trigger drug release within tumors [24]. Beyond accelerating drug release, previous studies have shown that certain linkers also enhance the self-assembly of prodrugs by strengthening intermolecular interactions [19,25]. These insights have broadened the functional scope of chemical linkers in prodrug-based delivery and sparked growing interest in the design of novel linker chemistries. However, rationally designing linkers that are both tumor-responsive and conducive to nanoassembly remains a significant challenge.

Precise modulation of intermolecular forces is a key driver in the formation of prodrug nanoassemblies, enabling stable self-assembly and efficient delivery [26,27]. Strategically designed prodrug molecules can introduce structural defects—such as specific functional group arrangements or steric configurations—that facilitate spontaneous formation of ordered nanostructures [19,28]. This process is governed by non-covalent interactions, including π–π stacking, hydrophobic effects, and hydrogen bonding, which collectively stabilize the assembled architecture [29,30]. Emerging evidence suggests that tuning hydrophobic and hydrogen bonding interactions can precisely control the morphology, size, and stability of nanoassemblies, thereby regulating drug release kinetics and influencing therapeutic efficacy [31,32,33]. For example, enhanced hydrophobicity may slow drug release and prolong circulation time, while engineered hydrogen bond networks can confer stimuli-responsive release behavior [34,35]. Despite the well-recognized role of π–π stacking in organic self-assembly systems [36], its contribution to prodrug nanoassemblies remains largely unexplored. How π–π interactions influence assembly behavior, shape nanoparticle properties, and impact in vivo delivery and antitumor activity remains unclear. Addressing this gap is essential to advancing structure–function understanding and guiding the design of next-generation anticancer nanomedicines.

**Scheme 1:**
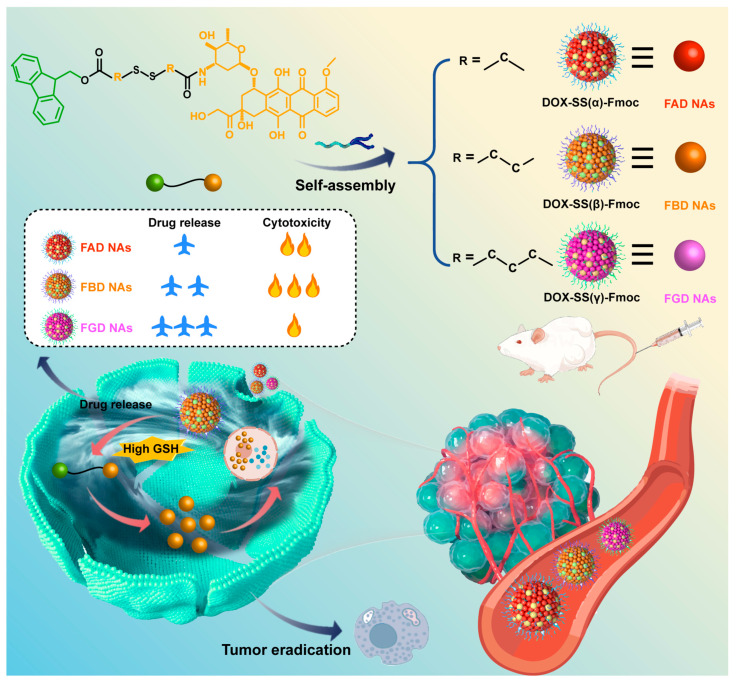
Redox-responsive π-conjugated prodrug nanoassemblies for cancer chemotherapy.

Guided by fundamental principles of molecular self-assembly, we engineered a next-generation DOX delivery system that couples π–π stacking with spatially programmed disulfide bonds (α, β, γ), achieving dual benefits of reduced carrier toxicity and controlled, efficient release. By leveraging the strong π–π stacking capability of Fmoc moieties to facilitate robust nanoassembly formation while systematically investigating the impact of disulfide bond positioning on both structural stability and redox-responsive drug release kinetics. As illustrated in Scheme 1, we first successfully synthesized three structurally optimized prodrug variants, DOX-SS(α)-Fmoc (FAD), DOX-SS(β)-Fmoc (FBD), and DOX-SS(γ)-Fmoc (FGD), which spontaneously self-assembled into stable carrier-free nanoassemblies (FAD NAs, FBD NAs, FGD NAs). Following intravenous administration, these nanoassemblies maintained structural stability to ensure tumor-targeted delivery while minimizing off-target toxicity of DOX. Upon internalization by tumor cells, the high intracellular GSH levels triggered their disassembly to release DOX, thereby exerting potent antitumor effects. Through comprehensive investigations encompassing prodrug synthesis and structural characterization, nanoassembly preparation and physicochemical evaluation, cellular uptake and cytotoxicity profiling, pharmacokinetic and biodistribution analyses, as well as systematic assessment of antitumor efficacy and preliminary safety evaluation in tumor-bearing models, we demonstrated that FBD NAs exhibited particularly stable tumor reduction-responsive drug release behavior and superior antitumor activity. This integrated approach not only yields critical insights into structure-activity relationships but also establishes a promising optimized DOX delivery platform with significant therapeutic potential.

## 2. Materials and Methods

### 2.1. Experimental Materials

Chemical reagents including HBTU, DIPEA, DMAP, DOX‧HCl, and Fmoc were sourced from Anaiji Chemical Co., Ltd. (Shanghai, China). DSPE-PEG_2K_ was acquired from AVTPharmaceutical Technology Co., Ltd. (Shanghai, China).All cell culture vessels (glass-bottom dishes, culture dishes, and plates) were obtained from NEST Biotechnology Co., Ltd. (Wuxi, China).

### 2.2. Design and Synthesis of Position-Specific Disulfide-Bridged DOX Prodrugs

We referred to the methods in the literature and synthesized three position-specific disulfide bridging DOX prodrugs (FAD, FBD, FGD) through a standardized three-step protocol [33,37]. First, dithiodiacids (2 mmol) were reacted with acetic anhydride (5 mL) at 25 °C for 2 h under N_2_, followed by toluene-assisted drying. The ints were then coupled with Fmoc (2 mmol) and DMAP (0.2 mmol) in dichloromethane (10 mL) at 25 °C for 12 h, purified by silica column chromatography (CH_2_Cl_2_: MeOH, 500:1). Finally, the Fmoc intermediates (0.5 mmol) were conjugated with DOX·HCl (0.5 mmol) using HBTU/DIPEA in DMF (10 mL) at 30 °C for 48 h. All products were purified by preparative high-performance liquid chromatography (HPLC, acetonitrile:water, 70:30) with yields >50% and characterized by mass spectrometry (MS) and NMR. The MS characterization of prodrugs was carried out by high-resolution electrospray ionization mass spectrometry (HR-ESI-MS) in the positive ion mode. The molecular structure and purity of the prodrug were confirmed by precisely measuring the molecular ion peaks ([M + H]^+^) and characteristic fragment peaks. The specific parameters are as follows: Scanning range: *m*/*z* 100–1500; drying temperature: 300 °C; capillary voltage: 3.5 kV; and collision energy: 20–40 eV (gradient optimized). The prodrugs were characterized by ^1^H NMR (400/600 MHz, DMSO-d_6_ with 0.03% TMS) at 25 °C, analyzing chemical shifts (δ 0–15 ppm), peak patterns (e.g., aromatic protons at 7.5–8.5 ppm), and integration ratios (e.g., Fmoc 9H protons) with acquisition parameters of 32–64 scans, 12 ppm spectral width, and 2 s relaxation delay.

### 2.3. Preparation of DOX Prodrug NAs

The three prodrug-based nanoassemblies (FAD NAs, FBD NAs, and FGD NAs) were synthesized using a standardized nanoprecipitation protocol [38,39]. In brief, individual prodrugs (1 mg) were initially dissolved in a 1:1 (*v*/*v*) THF/ethanol cosolvent system (200 μL total volume). This organic solution was then rapidly injected into deionized water (1 mL) under continuous magnetic stirring at 1050 rpm. For PEGylated variants, DSPE-PEG_2K_ (20% *w*/*w* relative to prodrug) was incorporated into the organic phase prior to aqueous injection. Following nanoassembly formation, residual organic solvents were removed by rotary evaporation at 30 °C under reduced pressure in 5 min.

Prior to full-scale production, comprehensive parameter optimization was conducted. This systematic evaluation assessed the impact of different organic solvents (THF, ethanol, methanol, acetonitrile, and various combinations) and stirring speeds (650–1950 rpm) on nanoassembly characteristics. Based on extensive characterization data, the THF/ethanol (1:1) cosolvent system combined with 1050 rpm stirring was identified as the optimal formulation condition, yielding nanoassemblies with the most favorable physicochemical properties.

For quality control, the prepared nanoassemblies were characterized using dynamic light scattering (Malvern Zetasizer, NanoZS, Malvern Co., Worcestershire, UK). Samples were appropriately diluted (50 μL nanoassemblies in 950 μL deionized water) and equilibrated for 120 s prior to measurement. We evaluated three critical parameters (hydrodynamic diameter, PDI, and zeta potential) with triplicate measurements to verify reproducibility.

### 2.4. In Vitro Stability Study of NAs

The colloidal stability of PEGylated FAD NAs, FBD NAs, and FGD NAs (0.5 mg/mL) was systematically evaluated under three conditions: (1) in PBS containing 10% FBS (pH 7.4) at 37 °C with shaking (100 rpm), with particle size monitored over 24 h; (2) during 30-day storage at 4 °C; and (3) the formulations were mixed with 100 mg sucrose in 2 mL tubes, vortexed for complete dissolution, transferred to vials, and lyophilized (−80 °C pre-freeze for 24 h followed by 24 h primary drying) in a freeze-dryer. Reconstitution with 1 mL of deionized water yielded homogeneous nanosuspensions, with stability assessed via pre-/post-lyophilization particle size changes and powder morphology. In addition, the encapsulation rates of the three nanoassemblies during the stability experiment were determined by ultrafiltration and HPLC.

### 2.5. Evaluation of Intermolecular Interaction

We conducted a comprehensive evaluation of the driving forces underlying nanoassemblies. The computational analysis was performed using the Yinfu Cloud platform (Guangzhou Yinfu Information Technology Co., Ltd., Guangzhou, China), which involved the following: (1) constructing energy-minimized three-dimensional prodrug structures (in mol2 format) with precise disulfide bond positioning and (2) performing small-molecule docking through conformational searching to characterize hydrogen bonding, π–π stacking, and hydrophobic interactions. Then three chemical disruptors were used to probe intermolecular forces in NAs: 50 mM NaCl (electrostatic interactions), 50 mM urea (hydrogen bonds), and 50 mM SDS (hydrophobic interactions). For each test, 900 μL of disruptor solution was mixed with 100 μL of PEGylated prodrug nanoassemblies in 2 mL tubes, then incubated (37 °C, 100 rpm, 3 h). Particle size and PDI were measured pre- and post-incubation. Significant increases confirmed force-specific disruption.

### 2.6. Drug Release

The release study was conducted using PBS (pH 7.4) containing 25% ethanol as the solvent, with three different dithiothreitol (DTT) concentrations (0, 10, and 100 μM). PEGylated FAD NAs, FBD NAs, and FGD NAs were introduced into 5 mL of release medium at a DOX-equivalent concentration of 200 μg/30 mL. The mixtures were then incubated in a constant-temperature shaker maintained at 37 °C with 100 rpm agitation. Aliquots were collected at specified time points: 0, 0.5, 1, 2, 4, 6, 8, 10, and 12 h for standard conditions; 0, 0.5, 1, and 4 h for FAD NAs in 10 μM DTT; and 0, 0.167, and 0.333 h for FAD NAs in 100 μM DTT. The released DOX prodrug was quantified by HPLC through peak area analysis.

### 2.7. Cell Culture

The mouse breast tumor cell line (4T1 cells), human non-small cell lung cancer (NSCLC) epithelial cell line (A549 cells), and murine fibroblasts (3T1 cells) were derived from the Cell Bank of the Chinese Academy of Sciences (Beijing, China). For cryopreservation, adherent cells at logarithmic growth phase (80% confluency) were trypsinized (0.25% trypsin, 1 min), centrifuged (1000 rpm, 5 min), and resuspended in freezing medium before storage at −80 °C. For recovery, frozen vials were rapidly thawed (37 °C, <1 min), centrifuged, and seeded in pre-warmed medium. Subculture followed the same trypsinization procedure, with a 1:5 split ratio into fresh medium. All cells were maintained at 37 °C/5% CO_2_ and passaged ≥3 times before experimental use to ensure stable morphology and proliferation.

### 2.8. Cellular Uptake

C-6-labeled nanoassemblies were prepared by co-dissolving DOX prodrug and C-6 (1.0 mg each, at a 9:1 molar ratio) in THF/ethanol (1:1, *v*/*v*), mixed with DSPE-PEG_2K_ (20% *w*/*w*), then nanoprecipitated into deionized water under stirring (1050 rpm, 3 min). Subsequently, organic solvents were removed by rotary evaporation (30 °C) to obtain nanoassemblies (200 μg/mL DOX equivalent, 20 μg/mL C-6). For uptake studies, 4T1 cells were treated with C-6 formulations (200 ng/mL) for 0.5/2 h. Qualitative analysis involved fixation, Hoechst staining, and confocal microscopy by DAPI/FITC channels (CLSM, C2, Nikon, Tokyo, Japan).

After incubation periods of 0.5 or 2 h, 4T1 cells were processed for flow cytometric analysis (BD FACSCalibur, BD Biosciences, San Jose, CA, USA) following the same protocol used for confocal microscopy. Cells were trypsinized, centrifuged, and resuspended in PBS (pH 7.4), then filtered into flow cytometry tubes for quantitative fluorescence measurement. All procedures were performed under light-protected conditions to prevent fluorophore degradation.

### 2.9. In Vitro Cytotoxicity Investigation

The cytotoxic effects of DOX Sol, FAD NAs, FBD Nas, and FGD NAs on tumor cells (4T1 and A549 cells) and normal cells (3T3 cells) were evaluated by MTT assay. Briefly, cells were enzymatically detached with trypsin, pelleted by centrifugation (1000 rpm, 5 min), and adjusted to 10^4^ cells/mL in complete medium. Cell suspensions (2000 cells/well) were seeded in 96-well plates and allowed to adhere for 15 h under standard culture conditions (37 °C, 5% CO_2_). Post-treatment, 20 μL of MTT reagent (5 mg/mL in PBS) was introduced per well. Following 4 h of metabolic conversion, the resulting formazan crystals were solubilized in 200 μL DMSO with orbital shaking (30 min, 300 rpm). Optical density was quantified at 490 nm using a microplate reader (ThermoFisher Scientific, Waltham, MA, USA), with cell viability expressed relative to untreated controls. Dose–response curves were generated via nonlinear regression to derive IC_50_ values. Strict aseptic techniques were maintained throughout all procedures.

### 2.10. Animal Housing

All experimental animals were purchased from Liaoning Changsheng Biotechnology Co., Ltd., with confirmed good health status, without genetic modification, and without any prior experimental procedures. Animal procedures were approved by the Institutional Animal Care and Use Committee of Shenyang Pharmaceutical University (Shenyang, China). The animal study protocol was approved by the Institutional Animal Ethics Committee (Approval Code: SYPU-2ACUC-S2024-0919-201; Approval Date: 19 September 2024). The study used adult male Sprague-Dawley (SD) rats (8–12 weeks old, ~200 g) and adult female BALB/c mice (6–8 weeks old, ~20 g). 20 SD rats and 68 BALB/c mice were housed under standard laboratory conditions with free access to food and water. Randomization of treatment order and cage positions was implemented, with blinded assessments to minimize handling and environmental biases.

### 2.11. Pharmacokinetics

A total of 20 SD rats were randomly allocated into four treatment groups: DiR Sol, FAD@DiR NAs, FBD@DiR NAs, and FGD@DiR NAs (*n* = 5 per group; the experimental unit is a single animal). Test compounds—either DiR Sol or DiR-labeled prodrug nanoassemblies—were administered via tail vein injection at a standardized DiR-equivalent dose of 2 mg/kg. Serial blood samples were obtained through the ophthalmic venous plexus at predetermined time points. Following collection, whole blood was immediately centrifuged to isolate plasma fractions. DiR concentrations in plasma samples were subsequently quantified using fluorescence measurement with a microplate reader, with appropriate standard curves and quality controls included in each assay run. No animals, experimental units, or data points were excluded, as all animals maintained normal health status, body weight, and experimental data without abnormalities.

### 2.12. Biodistribution

For tumor model establishment, 4T1 cells suspended in PBS (pH 7.4) were subcutaneously inoculated into the right flank of female BALB/c mice. When tumor volumes reached approximately 400 mm^3^, the 20 tumor-bearing mice were randomly allocated into four treatment groups: DiR Sol, FAD@DiR NAs, FBD@DiR NAs, and FGD@DiR NAs (*n* = 5) and intravenously administered either DiR Sol or DiR-labeled prodrug nanoassemblies at a DiR-equivalent dose of 2 mg/kg. Whole-body fluorescence imaging was conducted using an IVIS Spectrum imaging system (PerkinElmer IVIS Spectrum, Waltham, MA, USA) at predetermined time intervals. At the predetermined peak fluorescence time point, mice were euthanized, followed by excision of tumors and major organs (heart, liver, spleen, lungs, and kidneys) for ex vivo fluorescence imaging analysis. No animals, experimental units, or data points were excluded, as all animals maintained normal health status, body weight, and experimental data without abnormalities.

### 2.13. In Vivo Anti-Tumor Efficiency of Three NAs

When 48 4T1 tumor-bearing BALB/c mice reached a tumor volume of ~100 mm^3^, they were randomly divided into six groups (*n* = 5): Saline, DOX Sol, Doxil, FAD NAs, FBD NAs, and FGD NAs. Treatments were administered intravenously (DOX-equivalent dose: 10 mg/kg) every 3 days for five cycles (Day 0–12). Tumor volumes were calculated (V = a × b^2^/2, where a and b are the long and short diameters, respectively), and growth curves were plotted. On Day 14 (48 h post-final dose), mice were euthanized, and tumors were excised, weighed, and fixed for histopathological analysis (H&E staining, Servicebio, Wuhan). Serum biochemical parameters were analyzed to assess hepatorenal function. The initial group size was *n* = 8. During the experiment, animals from certain groups that failed to meet health standards were excluded, ensuring a final count of *n* = 5 per group for analysis. We also performed in vivo efficacy studies of FBD NAs at varying doses.

### 2.14. Processing of Animal Experimental Data

Animals were randomly assigned to control and treatment groups to prevent selection bias (e.g., avoiding unintentionally assigning healthier animals to one group), balance confounding variables (e.g., weight, age, litter effects), and meet statistical assumptions (e.g., independence of observations). For basic comparisons (e.g., *t*-tests or one-way ANOVA), *n* = 5 per group may provide ~80% power to detect a large effect size (e.g., Cohen’s *d* > 1.0) if variability is low. Healthy animals meeting predefined age, weight, and baseline health criteria were included in the study, while those exhibiting severe adverse effects (>20% weight loss) or technical failures were excluded, with all criteria established a priori. Data points identified as outliers (>2SD) through predefined statistical methods were excluded, and technical errors were discarded. The study adhered to these pre-established inclusion/exclusion protocols throughout the experimental and analytical processes. Animals were randomly assigned to control and treatment groups to prevent selection bias (e.g., avoiding unintentionally assigning healthier animals to one group), balance confounding variables (e.g., weight, age, litter effects), and meet statistical assumptions (e.g., independence of observations). Only the randomization coordinator knew group assignments during allocation but was excluded from subsequent procedures, while all other personnel (treatment administrators, outcome assessors, and statisticians) remained blinded throughout the study, using coded identifiers and analyzing de-identified data until final results were completed.

### 2.15. Statistical Analysis

All statistical analyses were conducted using GraphPad Prism version 9.0 (GraphPad Software, San Diego, CA, USA). Continuous variables are expressed as mean ± standard deviation. Between-group differences were analyzed by two-tailed Student’s t-test or one-way ANOVA followed by Tukey’s post hoc test, respectively, for two groups and multiple groups. Statistical significance was defined as *p* < 0.05, with the following levels indicated: * *p* < 0.05, ** *p* < 0.01, and *** *p* < 0.001.

## 3. Results and Discussion

### 3.1. Synthesis of Three Redox-Specific Prodrugs of DOX

**Figure 1:**
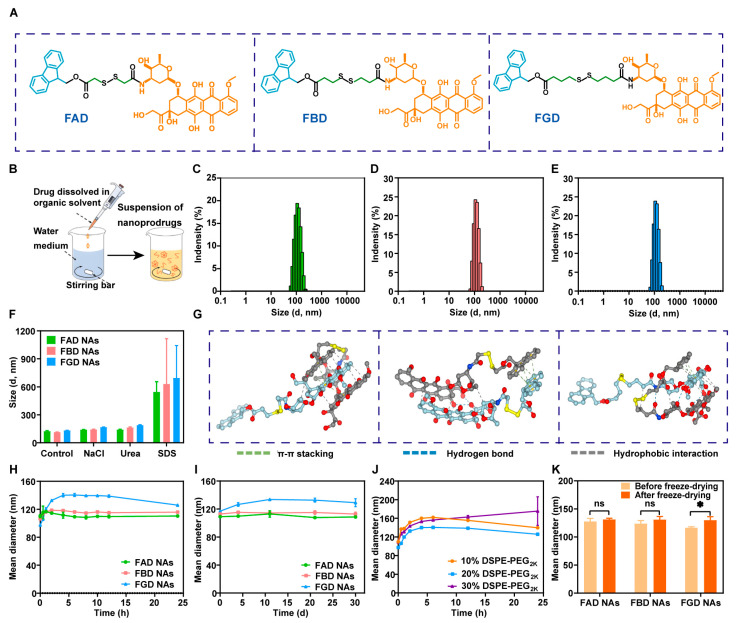
Fabrication and physicochemical characterization of DOX prodrug-based nanoassemblies. (**A**) The chemical structural formulas of FAD, FBD, and FGD. (**B**) Schematic diagram of the formulation process of one-step nano-precipitation method; particle size distribution images of the (**C**) FAD NAs, (**D**) FBD NAs, and (**E**) FGD NAs. (**F**) Particle size stability profiles of prodrug nanoassemblies (*n* = 3). (**G**) Molecular docking analysis of prodrug nanoassemblies. (**H**) Size evolution of prodrug NAs in PBS (pH 7.4, 10% FBS) was tracked (*n* = 3). (**I**) The long-term stability investigation results of three NAs (*n* = 3). (**J**) Colloidal stability of NAs with different PEG contents (*n* = 3). (**K**) The particle size of FAD, FBD, and FGD NAs before and after freeze-drying (*n* = 3) * *p* < 0.05, ns no significant.

To systematically investigate the impact of disulfide bond positioning on prodrug release kinetics and therapeutic efficacy, we designed three DOX prodrugs with distinct disulfide linkage modes at the α-, β-, and γ-positions relative to the amide bond (designated as FAD, FBD, and FGD, respectively). This structural optimization focused on the amino terminus of DOX, which represents an ideal site for chemical modification. The synthetic routes and chemical structures for these prodrugs are illustrated in Figure S1 and Figure 1A, with their chemical structures unequivocally confirmed by high-resolution mass spectrometry (HRMS) and ^1^H nuclear magnetic resonance (NMR) spectroscopy (Figure S2–S7).

### 3.2. Preparation and Characterization of NAs

Three disulfide-bridged DOX prodrug nanoassemblies (FAD NAs, FBD NAs, FGD NAs) were successfully prepared through optimized one-step nanoprecipitation (Figure 1B), incorporating 20% DSPE-PEG_2K_ for enhanced colloidal stability and prolonged circulation. Comprehensive formulation screening (Tables S1 and S2) established optimal preparation conditions. Characterization revealed monodisperse spherical nanoassemblies with a mean diameter of 110 ± 5 nm and exceptionally low polydispersity (PDI < 0.1) (Figure 1C–E, Table S3). The systems exhibited a zeta potential of −40 ± 2 mV (Table S3), ensuring electrostatic stabilization, along with FGD NAs exhibiting impressive drug loading capacity (45–50% *w*/*w*). TEM imaging (Figure S8) confirmed the uniform spherical morphology and successful self-assembly of all three configurations (α, β, and γ disulfide positions).

Mechanistic studies employing intermolecular force disruption (Figure 1F) demonstrated that while electrostatic (NaCl) and hydrogen bonding (urea) interactions contributed minimally to nanoassembly integrity, hydrophobic forces (SDS) played a predominant role in maintaining assembly stability. Complementary molecular docking simulations (Figure 1G) identified three key stabilizing interactions: (1) hydrogen bonding networks, (2) π–π stacking (particularly between Fmoc and the anthracycline moiety), and (3) hydrophobic effects, with the latter two contributing most significantly to molecular self-assembly.

Stability assessments under physiological conditions (10% FBS/PBS, pH 7.4) showed that FAD and FBD NAs maintained their original size distribution over 24 h, while FGD NAs reached a stable plateau at 130 nm (Figure 1H). Extended storage stability evaluations revealed consistent performance (Figure 1I), with the 20% PEG formulation demonstrating optimal characteristics (Figure 1J). In addition, all three nanoassemblies maintained high encapsulation efficiencies exceeding 90% during the stability study (Table S4), further demonstrating their excellent assembly stability. Lyophilization studies produced mechanically stable, orange-red cakes (Figure S9) that upon reconstitution showed minimal size variation for FAD NAs and FBD NAs and only a modest increase for FGD NAs (Figure 1K), confirming excellent lyophilization stability across all formulations.

### 3.3. Redox-Responsive Drug Release Behavior of NAs In Vitro

Malignant cells maintain substantially elevated intracellular GSH levels compared to their normal counterparts, creating a characteristically reductive tumor microenvironment. The disulfide bonds incorporated into DOX prodrugs undergo selective cleavage under these conditions, releasing DOX–thiol derivatives (DOX–SH). As established in previous studies, the release mechanism proceeds through sequential thiol–disulfide exchange reactions: (1) initial nucleophilic attack by GSH generates DOX-SSG, followed by (2) a second GSH-mediated cleavage that releases oxidized glutathione (GSSG) and yields the active DOX–SH species. The amide linkage in these prodrugs exhibits greater stability than ester bonds, preventing further hydrolysis to free DOX and maintaining DOX–SH as the terminal active form, which retains potent antitumor activity [17].

**Figure 2:**
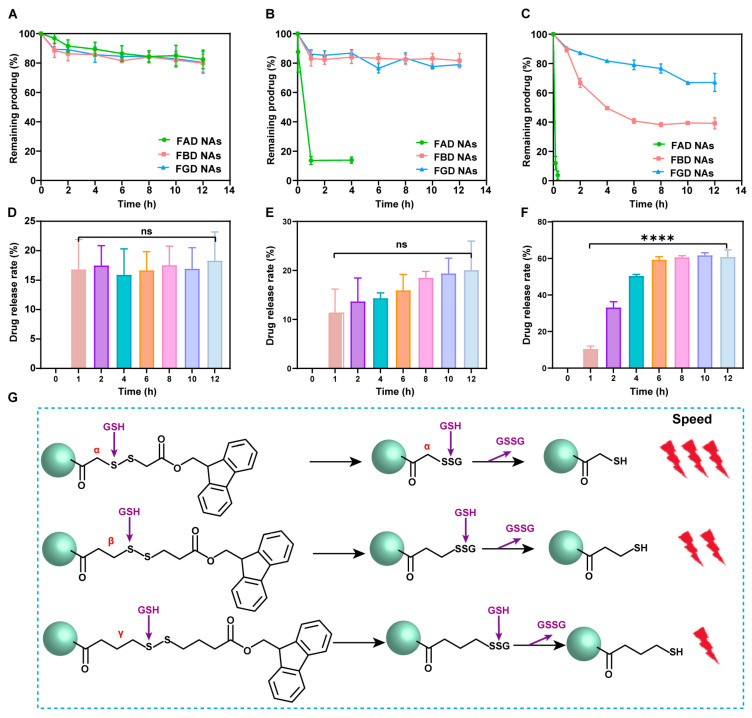
In vitro drug release of FAD, FBD, and FGD NAs in the presence of (**A**) 0 μM DTT, (**B**) 10 μM DTT, and (**C**) 100 μM DTT. The release amount of the parent drug DOX from FBD NAs in (**D**) 0 μm, (**E**) 10 μm, (**F**) 100 μm DTT. (**G**) Drug release mechanism of FAD NAs was triggered by DTT, FBD NAs, and FGD NAs. Data represent mean ± SEM; **** *p* < 0.0001, ns no significant, red lightning symbol, drug release speed.

The drug release behavior of DOX prodrug NAs was evaluated by monitoring prodrug degradation under simulated reductive conditions using DTT-containing media. In the absence of DTT, all three NA formulations (FAD NAs, FBD NAs, and FGD NAs) exhibited minimal degradation, demonstrating excellent stability (Figure 2A). However, distinct redox-responsive release profiles emerged with increasing DTT concentrations. FAD NAs showed the most rapid degradation, with over 80% and 90% of the prodrug released within 4 h at 10 μM and 100 μM DTT, respectively (Figure 2B,C). In contrast, FBD NAs displayed intermediate release kinetics, achieving 60% DOX release after 12 h at 100 μM DTT, as clearly illustrated in the time-dependent release profile (Figure 2D–F). FGD NAs demonstrated the slowest release, with less than 40% DOX liberated under the same conditions.

A clear concentration-dependent release pattern was observed, with accelerated degradation kinetics at higher DTT concentrations. As shown in Figure 2G, comparative analysis revealed a consistent structure-activity relationship, where reduction sensitivity followed the order: FAD NAs > FBD NAs > FGD NAs. This trend indicates that the drug release rate decreases with increasing carbon number in the disulfide linkage, highlighting the critical role of molecular structure in governing redox-responsive release behavior. Molecular docking simulations unexpectedly revealed that π–π stacking interactions serve as a dominant driving force for molecular assembly, highlighting the crucial regulatory role of Fmoc groups in this process—a key finding of our study.

### 3.4. Cellular Uptake

The three DOX prodrug variants, differing in disulfide bond positioning (α, β, and γ), all formed monodisperse nanoassemblies via self-assembly. Following PEGylation, these nanoassemblies exhibited enhanced colloidal stability and distinct redox-responsive drug release profiles, suggesting promising antitumor potential. To evaluate cellular uptake, we employed C-6 labeled formulations (C-6 Sol, FAD@C-6 NAs, FBD@C-6 NAs, and FGD@C-6 NAs) in 4T1 cells.

**Figure 3:**
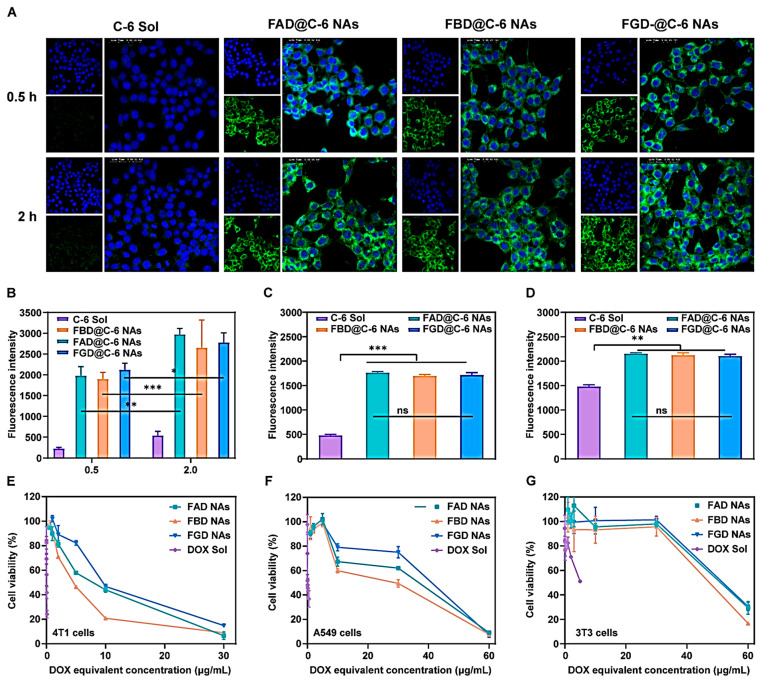
Cellular uptake and cytotoxicity. (**A**) Confocal laser scanning microscopy (CLSM) images of 4T1 cells incubated with C-6 Sol or C-6-labeled DOX prodrug NAs for 0.5 h and 2 h (scale bar = 10 μm). (**B**) Fluorescence quantification of CLSM images; flow cytometry measurements of 4T1 cells treated with C-6 Sol or C-6-labeled DOX prodrug NAs for 0.5 h (**C**) and 2 h (**D**) (*n* = 3). Cell viability of (**E**) 4T1, (**F**) A549, (**G**) 3T3 cells after being treated with various concentrations of DOX Sol and DOX prodrug NAs for 48 h (*n* = 3). Data represent mean ± SEM; *** *p* < 0.001, ** *p* < 0.01. * *p* < 0.05, ns no significant.

Confocal microscopy analysis demonstrated time-dependent cellular internalization, with significantly greater fluorescence intensity observed at 2 h compared to 0.5 h (Figure 3A,B). All three nanoformulations showed comparable uptake efficiency, each substantially exceeding that of free C-6 Sol, as evidenced by FITC fluorescence (green) relative to DAPI-stained nuclei (blue). Flow cytometric quantification corroborated these findings (Figure 3C,D), revealing (i) progressive accumulation over time (2 h > 0.5 h) and (ii) markedly enhanced cellular association of nanoassemblies versus free drug, with no significant differences among the three nanoformulations (*p* > 0.05).

### 3.5. Cytotoxicity

Previous in vitro characterization revealed distinct reduction-sensitive release profiles among the three prodrug nanoassemblies (FAD NAs, FBD NAs, and FGD NAs). While rapid drug release typically enhances cytotoxicity, the structural variations in the liberated DOX–thiol derivatives—with sulfhydryl groups at distinct positions (α, β, or γ) relative to the amide bond—may significantly influence their biological activity.

To elucidate these structure-activity relationships, we conducted comprehensive cytotoxicity evaluations using 4T1 cells, A549 cells, and 3T3 cells as normal cell controls. Comparative analysis demonstrated a consistent cytotoxic hierarchy in tumor cells (4T1/A549 cells): DOX Sol > FBD NAs > FAD NAs > FGD NAs (Figure 3E,F, Table S5). Notably, FBD NAs exhibited superior cytotoxicity compared to FAD NAs despite slower release kinetics, suggesting enhanced bioactivity of the β-position DOX–thiol derivative. In addition, FGD NAs exhibited the lowest cytotoxicity among all three cell lines, attributable to the stable assembly properties conferred by the γ-position disulfide bonds, which resulted in the slowest drug release rate and consequently minimal cellular toxicity. The drug release kinetics of NAs critically determine their therapeutic efficacy. While γ-disulfide bonds exhibit the strongest assembly stability with consequently slow drug release and minimal cytotoxicity, excessively rapid drug leakage (as seen with some linkers) compromises antitumor efficacy by disrupting the delivery-release balance. Notably, β-disulfide bonds demonstrate optimal intermediate release kinetics, achieving the ideal equilibrium between nanocarrier stability and efficient drug liberation for maximal therapeutic performance.

All nanoformulations showed significantly reduced cytotoxicity in 3T3 cells compared to malignant cells (Figure 3G), confirming their tumor-selective therapeutic potential. These findings collectively demonstrate that while release kinetics influence drug availability, the specific chemical architecture of the active metabolite ultimately determines cytotoxic potency, with the β-configuration proving most efficacious among the tested variants.

### 3.6. Pharmacokinetics and Biodistribution

**Figure 4:**
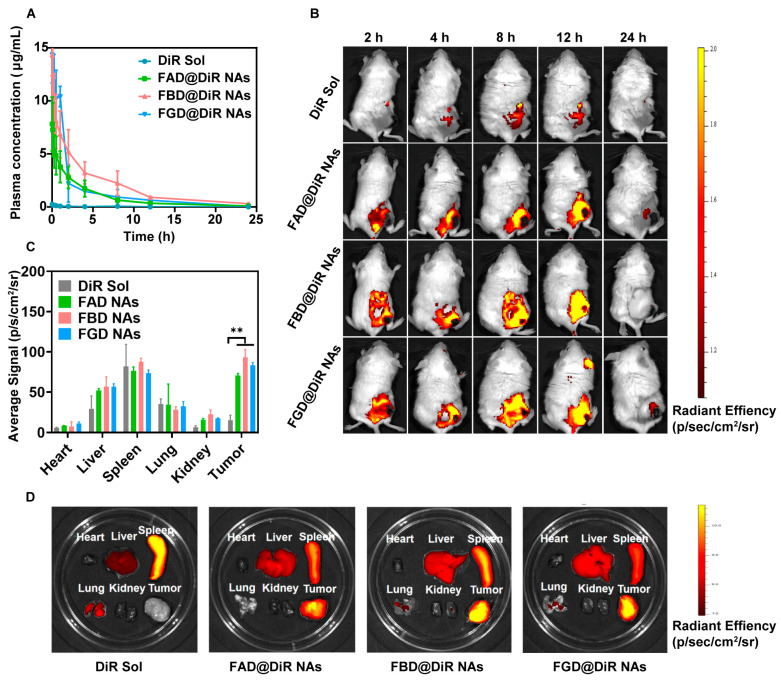
Pharmacokinetic and in vivo biodistribution. (**A**) Comparative pharmacokinetics of DiR Sol vs. DiR-labeled prodrug NAs (1 mg DiR eq./kg; *n* = 5). (**B**) Representative in vivo fluorescence images of 4T1-tumor-bearing mice following administration of DiR Sol or DiR-prodrug NAs (1 mg/kg; *n* = 5). (**C**) Quantitative results of fluorescence intensity in the major organs and tumors of 4T1 tumor-bearing BALB/c mice at 12 h (*n* = 3). (**D**) Ex vivo biodistribution analysis of DiR formulations in 4T1 tumor models. Data represent mean ± SEM; ** *p* < 0.01.

Our three strategically designed NAs with different disulfide bond positions demonstrated excellent stability, redox-responsive release of DOX–thiol derivatives, efficient tumor cell uptake, and significant in vitro cytotoxicity in previous studies. These promising properties necessitated further in vivo evaluation to assess their therapeutic potential. Following intravenous administration, nanotherapeutics must successfully reach tumor sites to exert pharmacological effects, raising the question of whether disulfide position variations would affect their pharmacokinetic behavior and tumor accumulation profiles. Due to its strong binding affinity with NAs, excellent fluorescence properties, minimal interference with in vivo fate, and low toxicity, DiR was selected as the fluorescent tracer to investigate the pharmacokinetic behavior of three DOX prodrug-based nanoassemblies. Pharmacokinetic analysis in SD rats revealed distinct plasma concentration-time curves for DiR Sol versus the three prodrug NAs (Figure 4A). This analysis was conducted under the premise of ensuring compliance with the standard curve requirements of DiR in rat plasma (Figure S10). While DiR Sol was rapidly cleared, PEGylation of the prodrug NAs created a protective hydration layer that minimized recognition by the reticuloendothelial system, resulting in significantly prolonged circulation times and greater AUC_0–24h_ values—properties conducive to enhanced tumor accumulation via the EPR effect. Comparative analysis showed FAD@DiR NAs exhibited the lowest AUC_0–24h_, likely due to excessive redox sensitivity leading to premature degradation by blood reductases. FGD@DiR NAs demonstrated suboptimal stability consistent with in vitro observations. In contrast, FBD@DiR NAs displayed balanced redox-responsiveness and stability, yielding superior pharmacokinetic performance with reduced degradation risk and optimal circulation characteristics among the three formulations (Table S6). These findings establish FBD@DiR NAs as the most promising candidate for further development.

Effective intratumoral drug accumulation represents a fundamental requirement for therapeutic efficacy, with drug concentration directly correlating with antitumor activity. Through quantitative in vivo fluorescence imaging, we evaluated the tumor-targeting capability of DiR-labeled prodrug nanoassemblies, where fluorescence intensity served as a direct indicator of tumoral drug enrichment. All three nanoformulations demonstrated superior tumor accumulation compared to DiR Sol (Figure 4B), reaching peak concentrations at 12 h post-administration. The tumor accumulation efficiency followed a distinct hierarchy: FBD@DiR NAs > FGD@DiR NAs > FAD@DiR NAs, directly mirroring their pharmacokinetic profiles. This correlation confirms that prolonged circulation time significantly enhances tumor deposition through the EPR effect. Comprehensive biodistribution analysis at 12 h (Figure 4C,D) revealed minimal tumor accumulation of DiR Sol, while the nanoassemblies showed substantial tumor enrichment, maintaining the same rank order. This enhanced targeting capability stems from synergistic effects of PEG-mediated prolonged circulation and optimized EPR characteristics, with FBD@DiR NAs exhibiting the most favorable profile for therapeutic applications.

### 3.7. In Vivo Antitumor Efficacy

Our designed DOX prodrug nanoassemblies with distinct disulfide linkages demonstrate four key advantages: (1) simple preparation with minimal excipients and high drug loading (~50%), (2) redox-responsive drug release in tumor microenvironments, (3) enhanced cellular uptake and cytotoxicity, and (4) prolonged circulation with tumor accumulation. While sharing these benefits, the α (FAD), β (FBD), and γ (FGD) configurations displayed differential release kinetics, cytotoxicity, pharmacokinetics, and biodistribution profiles due to their structural variations. This prompted a comprehensive evaluation of their therapeutic efficacy and safety in 4T1 tumor-bearing BALB/c mice.

**Figure 5:**
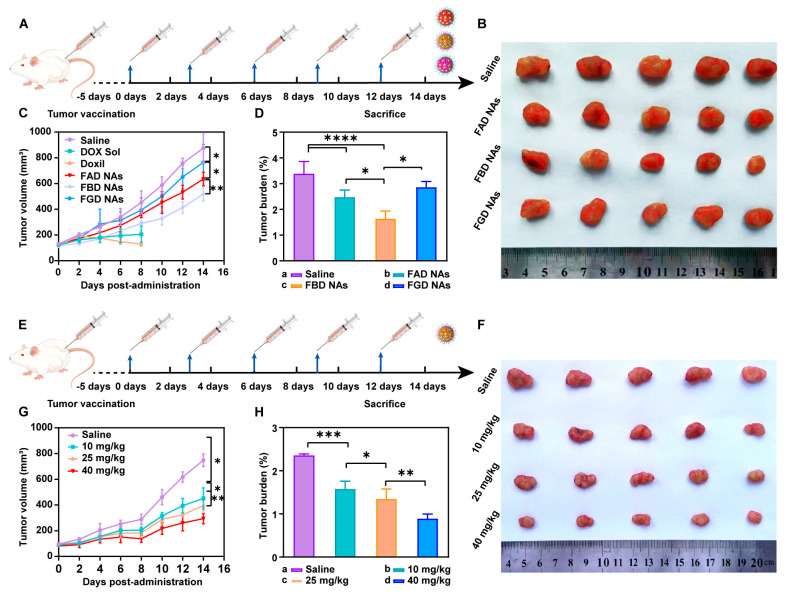
In vivo antitumor efficacy of prodrug nanoassemblies. (**A**) Treatment schedule of all NAs and control groups (*n* = 5). (**B**) Image of tumors after the last treatment (*n* = 5). (**C**) Tumor growth profiles treated with different formulations (*n* = 5). (**D**) Tumor burden after the last treatment. (*n* = 5). (**E**) Treatment schedule of different doses of FBD NAs (*n* = 5). (**F**) Image of tumors after the last treatment for different dose of FBD NAs and saline (*n* = 5). (**G**) Tumor growth profiles treated with different doses of FBD NAs (*n* = 5). (**H**) Tumor burden after the last treatment (*n* = 5). * *p* < 0.05, ** *p* < 0.01, *** *p* < 0.001, **** *p* < 0.0001, blue arrows. Injection time point.

Using 4T1 tumor-bearing BALB/c mice, we assessed antitumor efficacy through tumor volume measurements, burden rates, and histopathological analysis (H&E staining) across six treatment groups: Saline, DOX Sol, Doxil, and three prodrug nanoassemblies (FAD, FBD, and FGD NAs) at 10 mg/kg DOX-equivalent doses once every 3 days through injection for 5 times (Figure 5A). Initial tumor volumes ranged from 100 to 110 mm^3^. While conventional DOX formulations (DOX Sol and Doxil) proved lethal within 8 days, all nanoassemblies demonstrated significant tumor growth inhibition compared to saline controls (900 mm^3^ at day 14). FBD NAs emerged as the most effective formulation, showing superior tumor suppression (FBD > FAD > FGD; Figure 5B,C) that correlated with in vitro cytotoxicity data. This enhanced efficacy reflected FBD’s optimal combination of strong cytotoxicity, favorable pharmacokinetics, and efficient tumor accumulation, resulting in the lowest tumor burden rate (Figure 5D) and most pronounced histological evidence of tumor cell death (Figure S11). In contrast, FGD NAs exhibited reduced antitumor activity due to slower drug release kinetics and weaker cytotoxicity, while FAD NAs demonstrated intermediate performance. The consistent hierarchy across both in vitro and in vivo evaluations confirms the critical relationship between prodrug structure and therapeutic outcome.

Comprehensive safety evaluation in 4T1 tumor-bearing BALB/c mice demonstrated superior biocompatibility of the prodrug nanoassemblies (FAD, FBD, FGD NAs) compared to conventional DOX formulations. While DOX Sol and Doxil (10 mg/kg DOX-equivalent) caused 100% mortality within 8 days, all nanoassembly-treated groups maintained stable body weights (Figure S12) and showed normal hepatic (ALT, AST) and renal (CRE, BUN) function markers within physiological ranges (Figure S13). H&E analysis revealed intact tissue architecture in major organs (Figure S14), confirming excellent in vivo compatibility. These findings demonstrate that the prodrug nanoassemblies effectively mitigate DOX-associated systemic toxicity while maintaining antitumor efficacy, representing a significant therapeutic advancement.

Capitalizing on the established safety and efficacy profile of FBD NAs, we investigated its dose–response relationship in 4T1 tumor-bearing BALB/c mice at escalating DOX-equivalent doses (10, 25, and 40 mg/kg). The dosing regimen is shown in Figure 5E. Treatment initiation at 100 mm^3^ tumor volume revealed striking dose-dependent efficacy: while saline controls developed 800 mm^3^ tumors by day 14 (Figure 5F,G), all FBD NA groups showed significant growth inhibition, with tumor burden rates inversely correlating with dosage (Figure 5H). H&E analysis (Figure S15) demonstrated progressive therapeutic effects—from focal necrosis at 10 mg/kg to extensive tumor destruction at 40 mg/kg, characterized by complete cell death and architectural disintegration. These findings not only confirm dose-responsive antitumor activity of FBD NAs but also highlight their ability to safely deliver up to 4× the standard clinical DOX dose while maintaining superior tumor control. The preserved therapeutic index at high doses underscores exceptional clinical translation potential of FBD NAs as a next-generation anthracycline delivery platform.

Comprehensive safety assessment of FBD NAs at escalating doses (10–40 mg/kg DOX-equivalent) revealed excellent in vivo tolerability. All dose groups maintained stable body weights (Figure S16) and normal serum biochemistry (ALT, AST, CRE, BUN; Figure S17), with hepatic/renal markers consistently within physiological ranges. H&E examination (Figure S18) confirmed preserved architecture in major organs (heart, liver, spleen, lungs, kidneys) without evidence of inflammation or necrosis, even at the maximum 40 mg/kg dose. These findings demonstrate the exceptional safety profile of FBD NAs, maintaining full biocompatibility at doses 4-fold higher than conventional DOX’s maximum tolerated dose while delivering enhanced antitumor efficacy.

## 4. Conclusions and Discussion

In this study, we elucidate that the antitumor efficacy of precisely engineered prodrug nanoassemblies is intricately governed by the strategic placement of disulfide bonds in conjunction with π–π stacking interactions. These two factors synergistically modulate the drug release kinetics, thereby offering a solution to the long-standing delivery-release paradox that has plagued the field of nanomedicine. Our investigations reveal that robust π–π stacking interactions endow the prodrug nanoassemblies with exceptional self-driven assembly properties, facilitating their formation and stability. Concurrently, the systematic manipulation of disulfide bond locations within the prodrug architecture provides a precise means of controlling the drug liberation dynamics. Our experimental results clearly demonstrate position-dependent drug release kinetics, following the order of α > β > γ. Notably, the β-disulfide configuration emerges as the most clinically promising candidate, as it strikes an optimal balance between systemic stability and specific drug release, thus achieving the desired therapeutic efficacy.

This research represents an innovative integration of tumor reduction-responsive prodrug design with carrier-free nanoassembly technology. We have successfully developed three DOX prodrug nanoassemblies, namely FAD, FBD, and FGD NAs, which are distinguished by the different positions of disulfide bonds (α, β, and γ). To enhance the intermolecular interactions within these nanoassemblies, we incorporated 9-fluorenylmethanol as an aromatic conjugated side chain. Subsequently, we conducted a systematic evaluation of the impact of disulfide bond positioning on the properties of these nanoassemblies. The results indicate that the β-disulfide modified FBD NAs exhibit superior performance across multiple aspects. Firstly, they possess high stability, as evidenced by a particle size of approximately 110 nm, a zeta potential of −40 mV, and a drug loading capacity ranging from 45% to 50%. Secondly, they demonstrate controlled reduction-responsive release, with the release rate following the order of FAD > FBD > FGD. Thirdly, compared to their α- and γ-modified counterparts, FBD NAs exhibit superior cytotoxicity, more favorable pharmacokinetics (as indicated by the highest AUC_0–24h_ value), and enhanced tumor accumulation. In 4T1 tumor-bearing mice, FBD NAs not only achieved significantly enhanced antitumor efficacy compared to FAD and FGD NAs but also maintained safety at a dose of 40 mg/kg (DOX equivalent), effectively addressing the toxicity limitations associated with conventional DOX formulations such as solutions and liposomes. These findings underscore the critical importance of disulfide bond positioning in prodrug design, with β-site modification uniquely optimizing the stability and release kinetics of the nanoassemblies. Overall, this study establishes a robust prodrug design strategy and a comprehensive structure-activity relationship framework, which hold great promise for advancing the clinical translation of tumor-targeted nanomedicines.

## Data Availability

The original contributions presented in this study are included in the article and supplementary material. Further inquiries can be directed to the corresponding authors.

## Supplementary Materials

The following supporting information can be downloaded at: https://www.mdpi.com/article/10.3390/pharmaceutics17091162/s1, Figure S1: The synthetic route of FAD, FBD, and FGD; Figure S2: Mass spectrum of FAD; Figure S3: ^1^H NMR spectrum of FAD; Figure S4: Mass spectrum of FBD; Figure S5: ^1^H NMR spectrum of FBD; Figure S6: Mass spectrum of FGD; Figure S7: ^1^H NMR spectrum of FGD; Figure S8: The transmission electron microscope image of (A) FAD, (B) FBD, and (C) FGD NAs. Scale bar represents 200 nm; Figure S9: Photographs of FAD, FBD, and FGD NAs before (A) and after (B) resolution; Figure S10: Standard curve of DiR in rat plasma; Figure S11: H&E staining images of tumor sections after the last treatment. Scale bar = 100 μm; Figure S12: Body weight changes in BALB/c mice bearing 4T1 tumor xenografts during treatment with different formulations (*n* = 5); Figure S13: Hepatorenal function parameters of mice bearing 4T1 tumor xenografts after the last treatment (*n* = 3); Figure S14: H&E staining images of the major organs of mice bearing 4T1 tumor xenografts after the last treatment. Scale bar = 100 μm; Figure S15: H&E staining images of tumor sections after the last treatment. Scale bar = 100 μm; Figure S16: Body weight changes in BALB/c mice bearing 4T1 tumor xenografts during treatment with different doses of FBD NAs (*n* = 5); Figure S17: Hepatorenal function parameters of mice bearing 4T1 tumor xenografts after the last treatment (*n* = 3); Figure S18: H&E staining images of the major organs of mice bearing 4T1 tumor xenografts after the last treatment. Scale bar = 100 μm; Table S1: Characterization of FAD, FBD, and FGD NAs with different solvents (*n* = 3); Table S2: Characterization of FAD, FBD, and FGD NAs with different rotate speeds (*n* = 3); Table S3: The characteristics of FAD, FBD, and FGD NAs; Table S4: Table S4. Encapsulation rates at different time points of FAD, FBD and FGD NAs (*n* = 3). Table S5: IC_50_ values of DOX Sol and DOX prodrug NAs against 4T1, A549, and 3T3 cells; Table S6: Pharmacokinetic parameters of DiR Sol and DiR-labeled DOX prodrug NAs (*n* = 5).
