# Supplementary material for: Redox-Responsive π-Conjugated Prodrug Nanoassemblies for Cancer Chemotherapy
Source: Pharmaceutics. 2025 Sep 4;17(9):1162. doi: 10.3390/pharmaceutics17091162 (PMC12473931; doi:10.3390/pharmaceutics17091162)
Supplement: Supplementary file 1 [file pharmaceutics-17-01162-s001.zip › pharmaceutics-3801327-supplementary.pdf]

Redox-Responsive π-Conjugated Prodrug Nanoassemblies for Cancer Chemotherapy

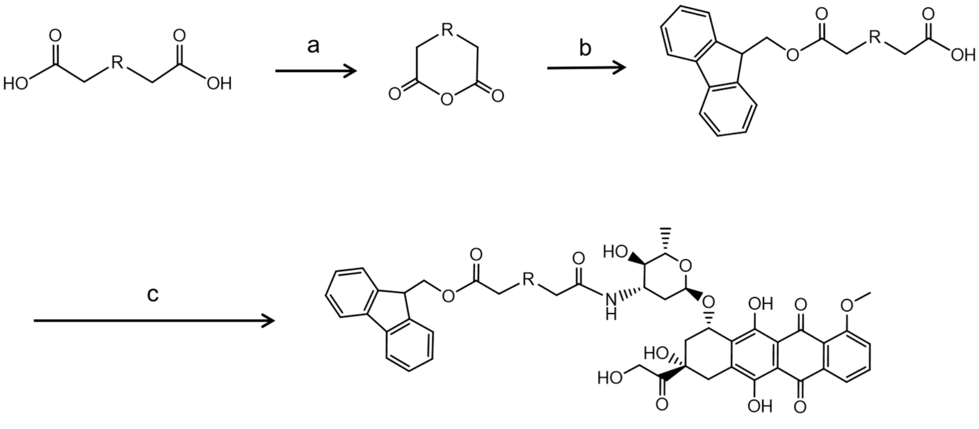

R=C1,α；R=C2,β；R=C3,γ

Figure S1. The synthetic route of FAD, FBD and FGD. (a) Acetic anhydride, 25°C, 2 h; (b) 9-fluorenyl alcohol, DMAP, 25°C, 12 h; (c) HBTU, DIPEA, 0°C, 2 h; DIPEA, DOX·HCl, 30°C, 48 h.

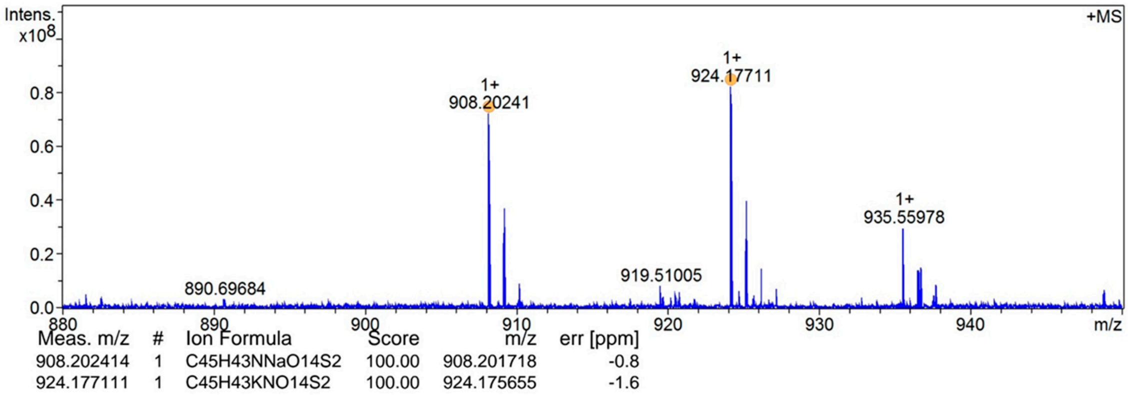

Figure S2. Mass spectrum of FAD. $[M + K]^+ = 924.17711$.

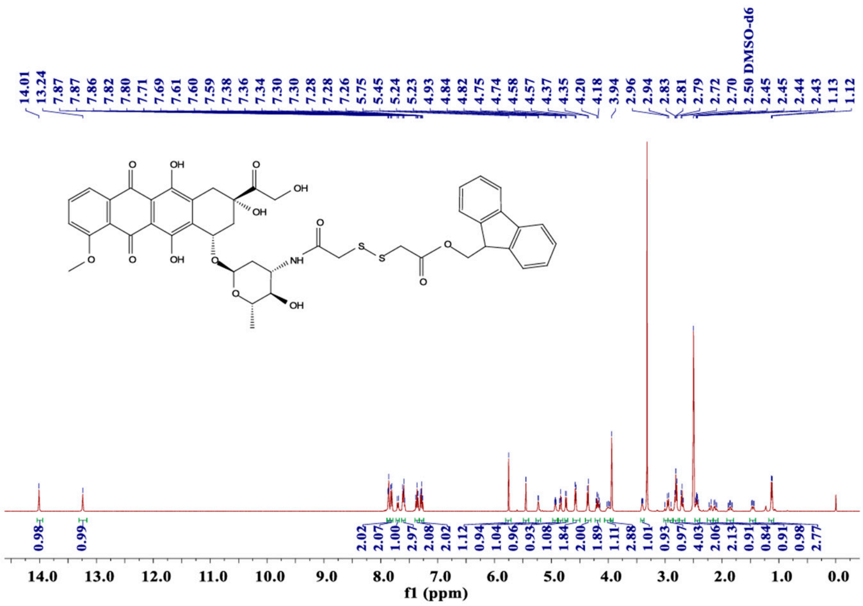

Figure S3. ^{1}H NMR spectrum of FAD. ^{1}H NMR (400 MHz, DMSO-d6) δ 14.01 (s, 1H), 13.24 (s, 1H), 7.89 – 7.85 (m, 2H), 7.81 (d, J = 7.5 Hz, 2H), 7.70 (d, J = 8.1 Hz, 1H), 7.64 – 7.56 (m, 3H), 7.36 (t, J = 7.5 Hz, 2H), 7.28 (dd, J = 8.0, 6.8 Hz, 2H), 5.75 (s, 1H), 5.45 (s, 1H), 5.23 (d, J = 3.1 Hz, 1H), 4.93 (dd, J = 5.6, 3.6 Hz, 1H), 4.84 (t, J = 5.9 Hz, 1H), 4.74 (d, J = 6.0 Hz, 1H), 4.58 (d, J = 6.0 Hz, 2H), 4.36 (d, J = 6.8 Hz, 2H), 4.22 – 4.13 (m, 2H), 4.06 – 3.96 (m, 1H), 3.94 (s, 3H), 3.40 (dd, J = 6.8, 2.1 Hz, 1H), 2.98 (d, J = 18.3 Hz, 1H), 2.92 (d, J = 18.2 Hz, 1H), 2.81 (t, J = 6.9 Hz, 4H), 2.70 (t, J = 6.7 Hz, 2H), 2.48 – 2.40 (m, 2H), 2.21 (d, J = 14.3 Hz, 1H), 2.12 (dd, J = 14.3, 5.6 Hz, 1H), 1.86 (td, J = 12.8, 3.9 Hz, 1H), 1.46 (dd, J = 12.4, 4.5 Hz, 1H), 1.13 (d, J = 6.4 Hz, 3H).

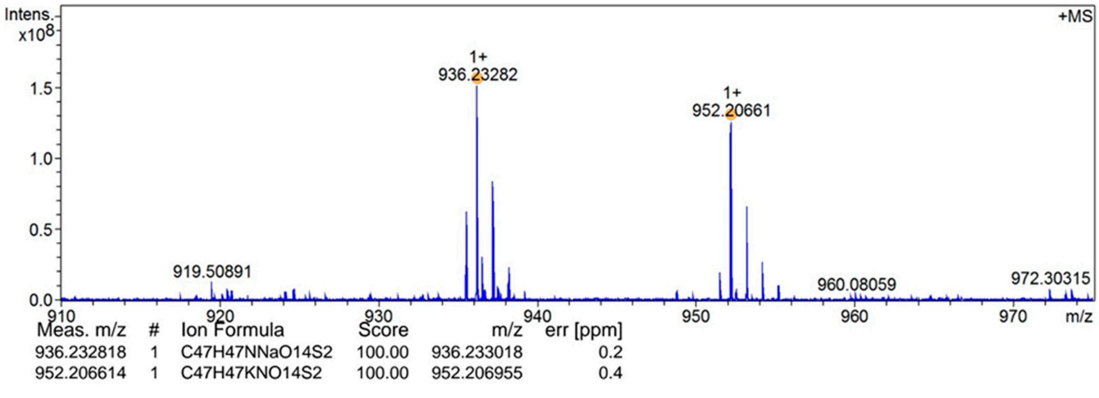

Figure S4. Mass spectrum of FBD. $[M + K]^{+}$ = 952.21

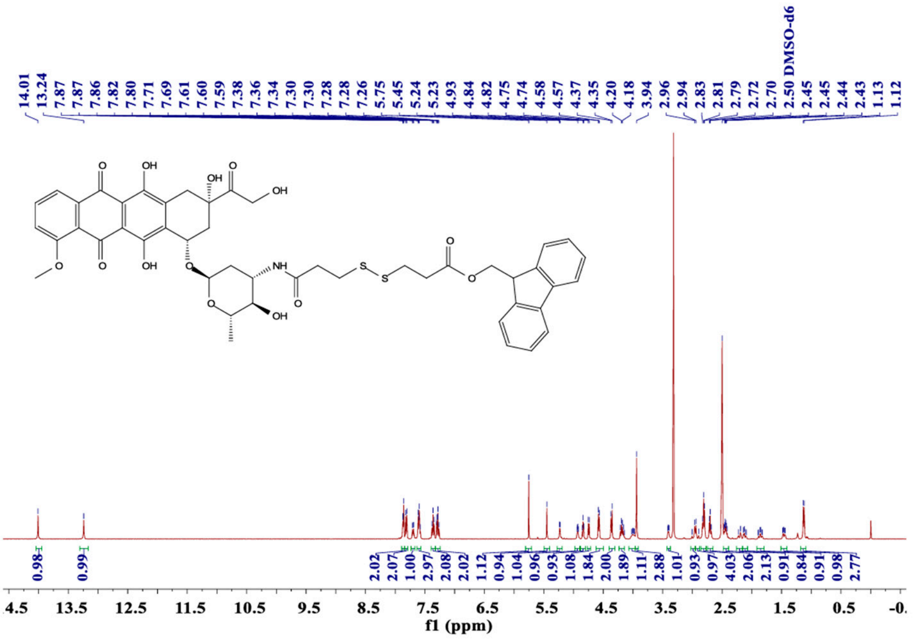

Figure S5. ^{1}H NMR spectrum of FBD. (400 MHz, DMSO-d6) δ 14.01 (s, 1H), 13.24 (s, 1H), 7.91 – 7.85 (m, 2H), 7.83 (d, J = 7.6 Hz, 2H), 7.65 – 7.52 (m, 4H), 7.37 (t, J = 7.5 Hz, 2H), 7.29 (t, J = 7.4 Hz, 2H), 5.44 (s, 1H), 5.23 (d, J = 3.6 Hz, 1H), 4.93 (t, J = 4.3 Hz, 1H), 4.84 (t, J = 5.9 Hz, 1H), 4.73 (d, J = 6.0 Hz, 1H), 4.58 (d, J = 6.0 Hz, 2H), 4.41 (d, J = 6.4 Hz, 2H), 4.26 – 4.11 (m, 2H), 4.04 – 3.97 (m, 1H), 3.95 (s, 3H), 3.40 (d, J = 4.9 Hz, 1H), 2.98 (d, J = 18.5 Hz, 1H), 2.92 (d, J = 18.4 Hz, 1H), 2.61 (t, J = 7.2 Hz, 2H), 2.56 – 2.51 (m, 2H), 2.34 (t, J = 7.2 Hz, 2H), 2.28 – 2.06 (m, 4H), 1.89 – 1.81 (m, 1H), 1.81 – 1.67 (m, 4H), 1.44 (dd, J = 12.9, 4.5 Hz, 1H), 1.13 (d, J = 6.4 Hz, 3H).

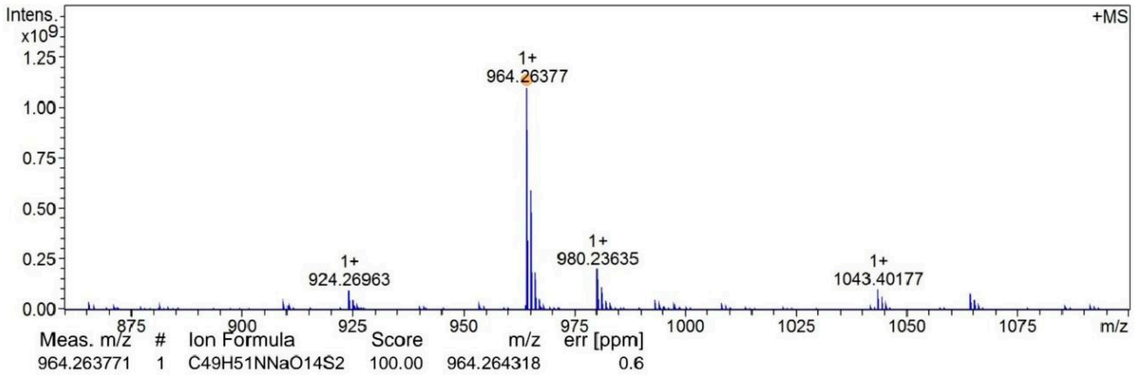

Figure S6. Mass spectrum of FGD. $[M + Na]^{+}$ = 964.26377

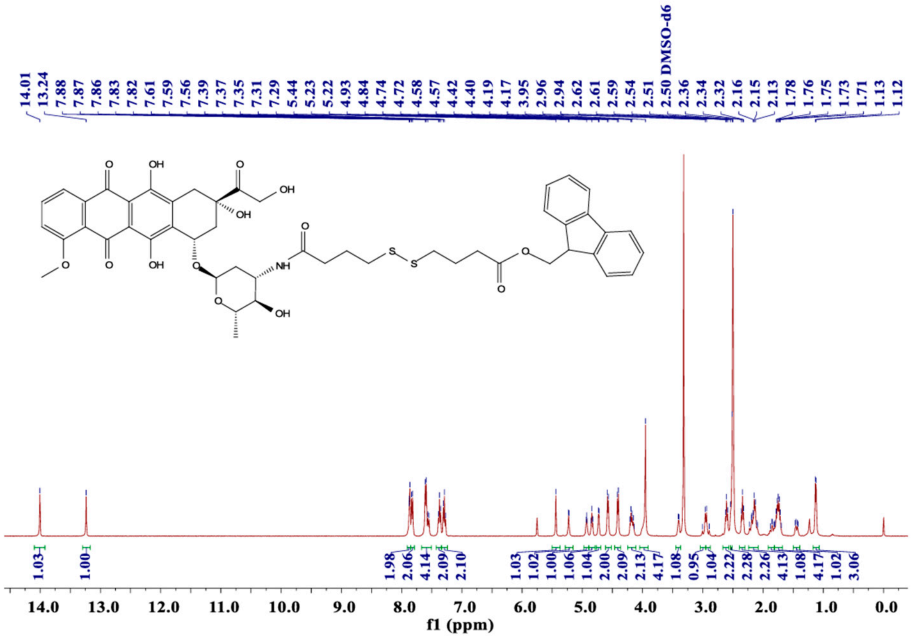

Figure S7. ^{1}H NMR spectrum of FGD. ^{1}H NMR (400 MHz, DMSO-d6) δ 14.01 (s, 1H), 13.24 (s, 1H), 7.91 – 7.85 (m, 2H), 7.83 (d, J = 7.6 Hz, 2H), 7.65 – 7.52 (m, 4H), 7.37 (t, J = 7.5 Hz, 2H), 7.29 (t, J = 7.4 Hz, 2H), 5.44 (s, 1H), 5.23 (d, J = 3.6 Hz, 1H), 4.93 (t, J = 4.3 Hz, 1H), 4.84 (t, J = 5.9 Hz, 1H), 4.73 (d, J = 6.0 Hz, 1H), 4.58 (d, J = 6.0 Hz, 2H), 4.41 (d, J = 6.4 Hz, 2H), 4.26 – 4.11 (m, 2H), 4.04 – 3.97 (m, 1H), 3.95 (s, 3H), 3.40 (d, J = 4.9 Hz, 1H), 2.98 (d, J = 18.5 Hz, 1H), 2.92 (d, J = 18.4 Hz, 1H), 2.61 (t, J = 7.2 Hz, 2H), 2.56 – 2.51 (m, 2H), 2.34 (t, J = 7.2 Hz, 2H), 2.28 – 2.06 (m, 4H), 1.89 – 1.81 (m, 1H), 1.81 – 1.67 (m, 4H), 1.44 (dd, J = 12.9, 4.5 Hz, 1H), 1.13 (d, J = 6.4 Hz, 3H).

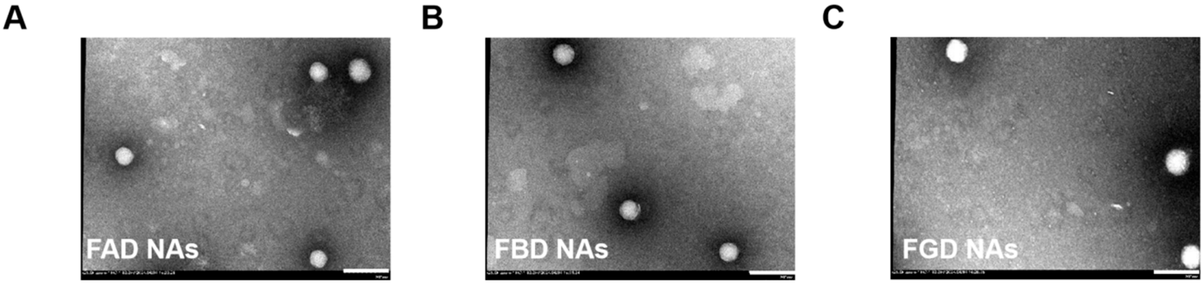

Figure S8. The transmission electron microscope image of (A) FAD, (B) FBD and (C) FGD NAs. Scale bar represents 200 nm.

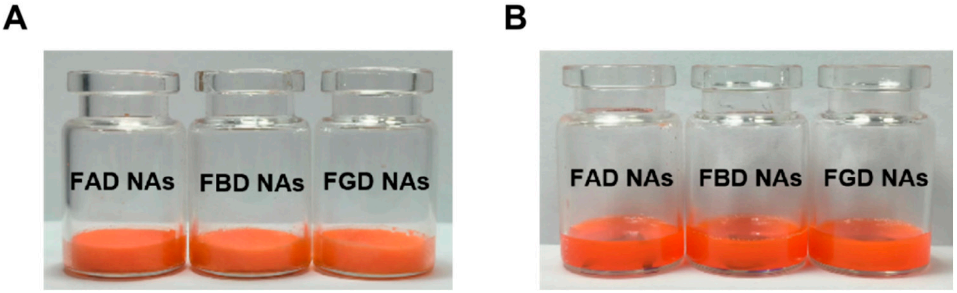

Figure S9. Photographs of FAD, FBD, and FGD NAs before (A) and after (B) resolution.

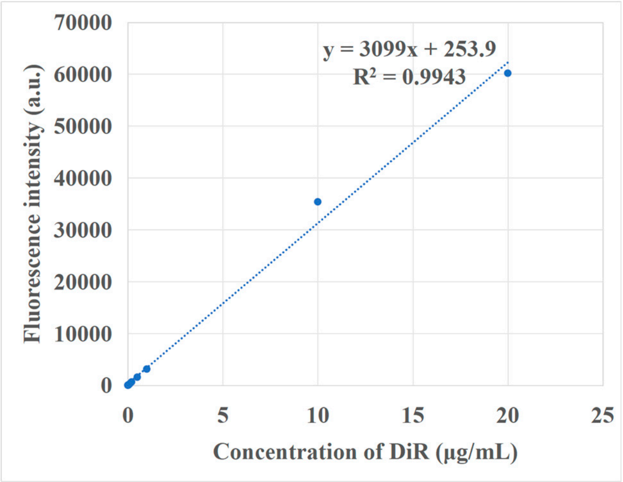

Figure S10. Standard curve of DiR in rat plasma.

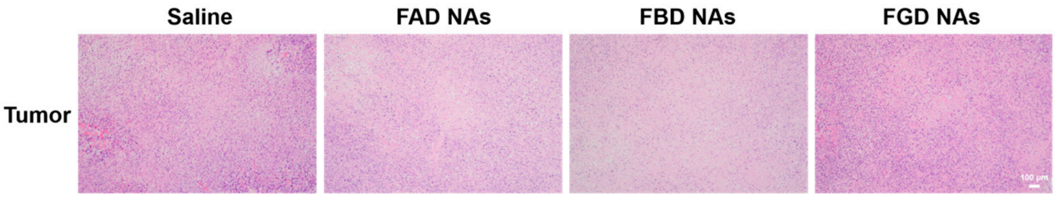

Figure S11. H&E staining images of tumor sections after the last treatment. Scale bar=100 μm

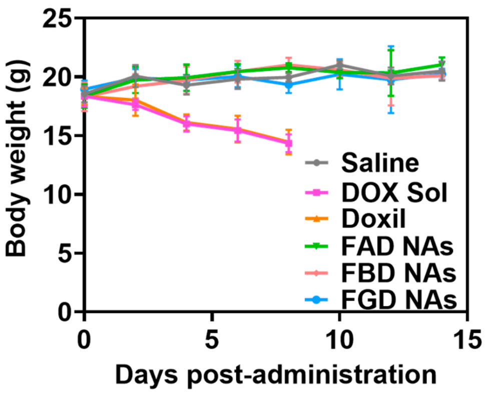

Figure S12. Body weight changes in BALB/c mice bearing 4T1 tumor xenograft during treatment with different formulations (n = 5).

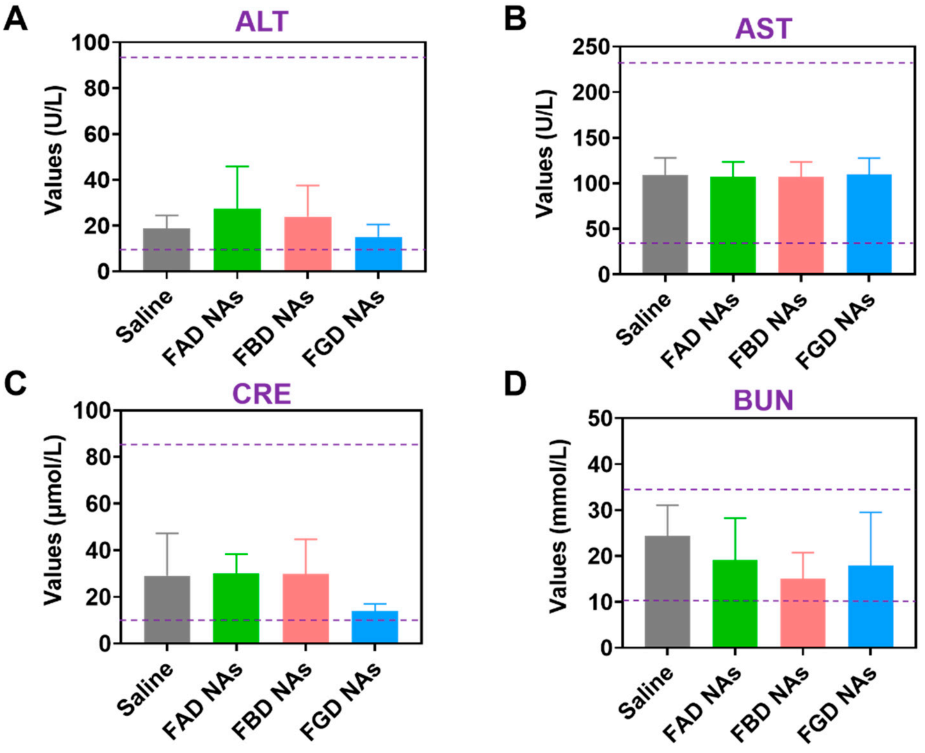

Figure S13. Hepatorenal function parameters of mice bearing 4T1 tumor xenografts after the last treatment (n = 3). (A) ALT. (B) AST. (C) CRE. (D) BUN. (The purple dashed line represents the maximum value and the minimum value.)

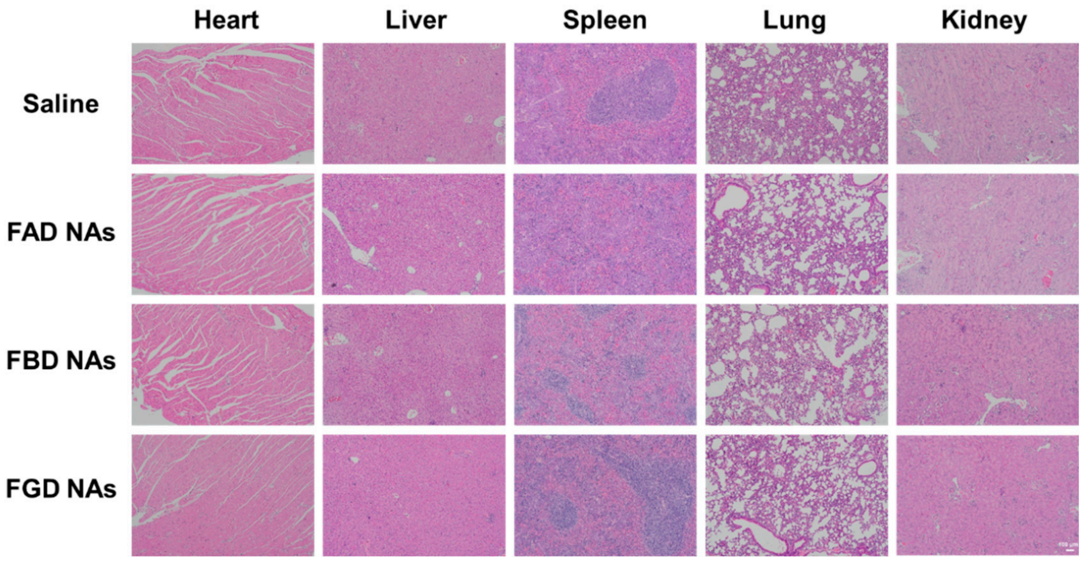

Figure S14. H&E staining images of the major organs of mice bearing 4T1 tumor xenografts after the last treatment. Scale bar=100 μm.

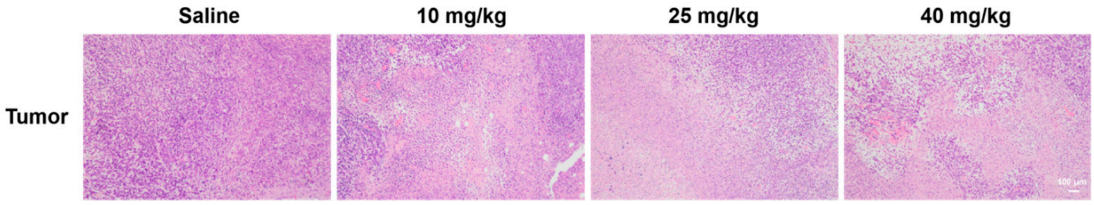

Figure S15. H&E staining images of tumor sections after the last treatment. Scale bar=100 μm.

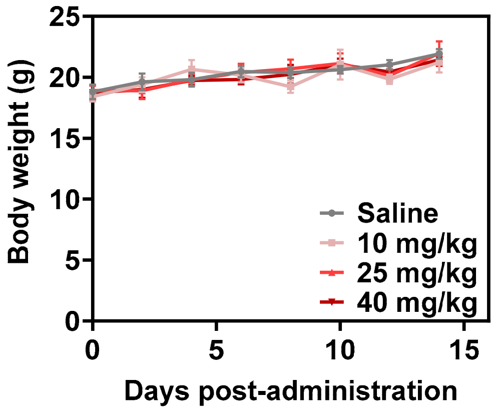

Figure S16. Body weight changes in BALB/c mice bearing 4T1 tumor xenograft during treatment with different doses of FBD NAs (n=5).

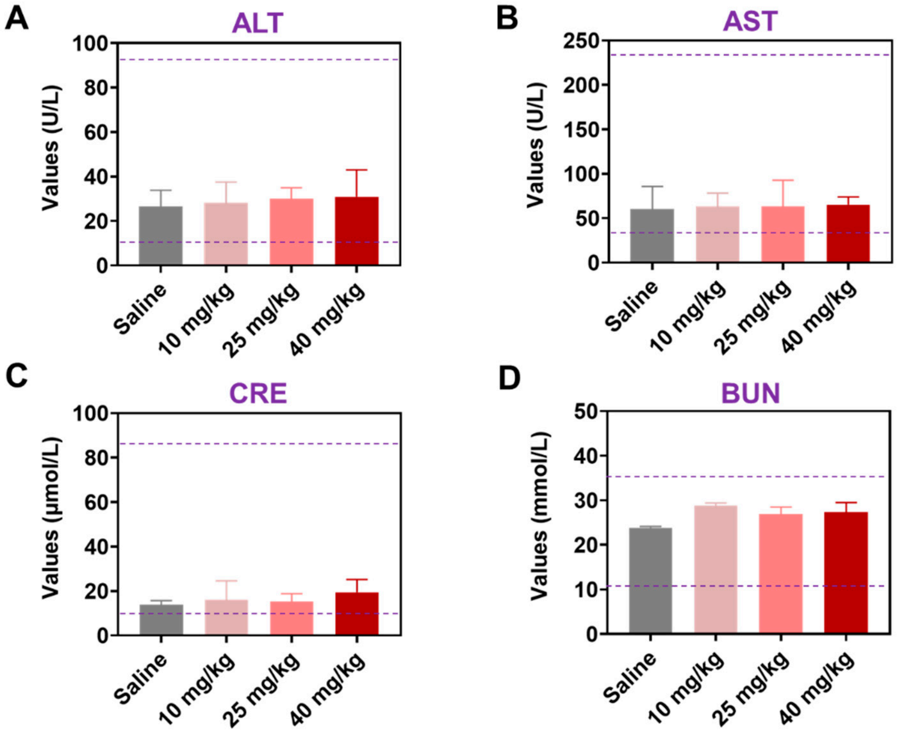

Figure S17. Hepatorenal function parameters of mice bearing 4T1 tumor xenografts after the last treatment (n=3). (A) ALT. (B) AST. (C) CRE. (D) BUN. (The purple dashed line represents the maximum value and the minimum value.)

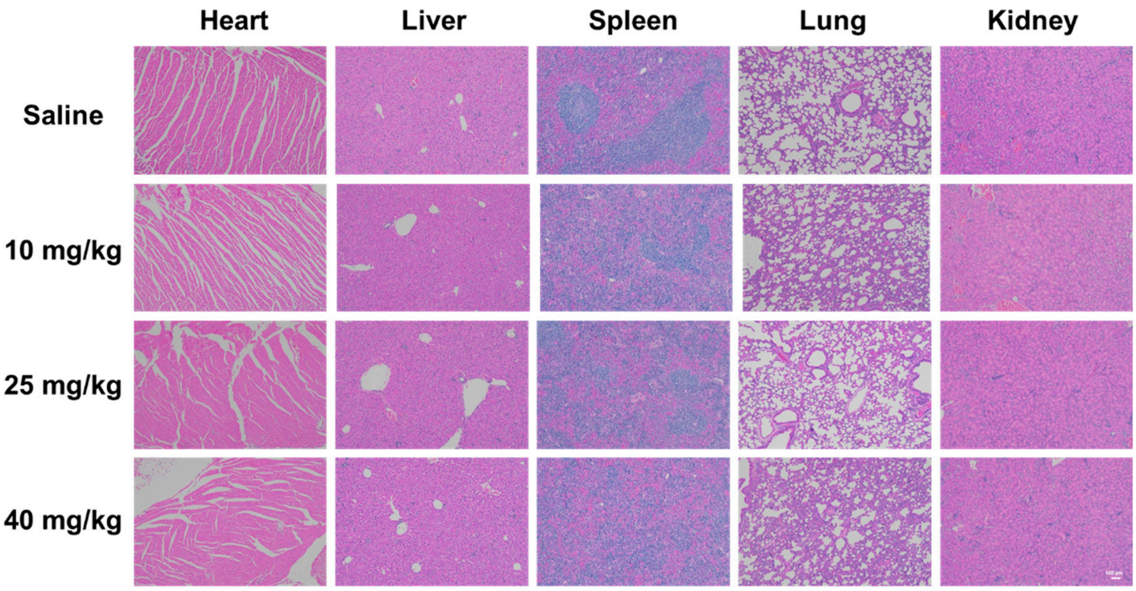

Figure S18. H&E staining images of the major organs of mice bearing 4T1 tumor xenografts after the last treatment. Scale bar=100 μm.

Table S1. Characterization of FAD, FBD, and FGD NAs with different solvents (n = 3).

Organic solvent	Formulations	Size (nm)	PDI
Tetrahydrofuran	FAD NAs	133.4 ± 3.4	0.01 ± 0.02
	FBD NAs	130.7 ± 2.6	0.09 ± 0.01
	FGD NAs	131.0 ± 1.0	0.08 ± 0.05
Tetrahydrofuran-Absolute ethyl alcohol（1:1, V/V）	FAD NAs	104.2 ± 0.6	0.10 ± 0.04
	FBD NAs	112.8 ± 1.3	0.10 ± 0.01
	FGD NAs	109.3 ± 1.6	0.09 ± 0.04

Table S2. Characterization of FAD, FBD, and FGD NAs with different rotate speeds (n = 3).

Rotate speed (rpm)	Formulations	Size (nm)	PDI
650	FAD NAs	127.1 ± 2.2	0.03 ± 0.01
	FBD NAs	128.1 ± 2.9	0.08 ± 0.04
	FGD NAs	108.2 ± 2.7	0.02 ± 0.01
1050	FAD NAs	104.2 ± 0.6	0.10 ± 0.04
	FBD NAs	112.8 ± 1.3	0.10 ± 0.01
	FGD NAs	109.3 ± 1.6	0.09 ± 0.04
1500	FAD NAs	103.0 ± 0.4	0.15 ± 0.06
	FBD NAs	96.2 ± 0.2	0.12 ± 0.14
	FGD NAs	95.3 ± 0.9	0.03 ± 0.03
1950	FAD NAs	104.5 ± 2.1	0.16 ± 0.04
	FBD NAs	84.3 ± 1.0	0.18 ± 0.04
	FGD NAs	99.2 ± 2.6	0.15 ± 0.03

Table S3. The characteristics of FAD, FBD, and FGD NAs.

Nanoassemblies	Size[a)] (nm)	PDI[b)]	*Zeta* potential[a)] (mV)	Drug loading (%)
FAD NAs	111.4 ± 1.9	0.06 ± 0.03	-44.51 ± 1.41	49.07
FBD NAs	115.3 ± 0.5	0.05 ± 0.03	-37.55 ± 1.48	47.61
FGD NAs	118.5 ± 1.6	0.04 ± 0.02	-40.77 ± 0.10	46.19

[a)] Mean diameter and *Zeta* potential of nanoassemblies were determined by Zetasizer Nano ZS. [b)] Polydispersity index of nanoassemblies.

Table S4. Encapsulation rates at different time points of FAD, FBD and FGD NAs (n = 3).

Time	FAD NAs	FBD NAs	FGD NAs
2 h	98.8% ± 2.12	97.5% ± 0.32	97.2% ± 14.5
4 h	96.3% ± 43.6	97.2% ± 8.24	97.1% ± 5.14
6 h	96.4% ± 1.65	95.7% ± 32.5	96.7% ± 23.5
12 h	95.4% ± 4.83	95.1% ± 2.41	95.6% ± 3.12
24 h	94.7% ± 12.7	95.0% ± 19.2	95.4% ± 7.92

Data are presented as mean ± SD (n = 3).

Table S5. IC_{50} values of DOX Sol and DOX prodrug NAs against 4T1, A549, and 3T3 cells.

Cell lines	DOX Sol	FAD NAs	FBD NAs	FGD NAs
4T1	0.03	6.89	4.43	11.32
A549	0.10	27.26	18.85	34.33
3T3	4.50	77.96	56.96	82.58

Table S6. Pharmacokinetic parameters of DiR Sol and DiR-labeled DOX prodrug NAs (n = 5).

**Formulations**	**Determined**	**$AUC_{0\text{-}24\,h}$ [a)]**	**$t_{1/2}$ [b)]**	**$MRT_{0\text{-}24\,h}$ [c)]**
DiR Sol	DiR	0.47 ± 0.18	4.53 ±3.44	3.37 ± 1.42
FAD@DiR NAs	DiR	22.24 ± 6.68	4.35 ± 1.12	4.66 ± 0.66
FBD@DiR NAs	DiR	47.79 ± 12.08	6.12 ± 1.03	5.56 ± 0.38
FGD@DiR NAs	DiR	32.90 ± 5.68	3.46 ± 0.85	3.90 ±1.01

[a)] Area under the plasma concentration–time curve (μg/mL*h); [b)] half-life (h); [c)] mean residence time (h).
